# Automated Imaging, Tracking, and Analytics Pipeline for Differentiating Environmental Effects on Root Meristematic Cell Division

**DOI:** 10.3389/fpls.2019.01487

**Published:** 2019-11-19

**Authors:** Eli Buckner, Imani Madison, Hsuan Chou, Anna Matthiadis, Charles E. Melvin, Rosangela Sozzani, Cranos Williams, Terri A. Long

**Affiliations:** ^1^Electrical and Computer Engineering Department, North Carolina State University, Raleigh, NC, United States; ^2^Plant and Microbial Biology Department, North Carolina State University, Raleigh, NC, United States

**Keywords:** light sheet imaging, image analysis, cell cycle progression, heat stress and iron deficiency stresses, combined stresses

## Abstract

Exposure of plants to abiotic stresses, whether individually or in combination, triggers dynamic changes to gene regulation. These responses induce distinct changes in phenotypic characteristics, enabling the plant to adapt to changing environments. For example, iron deficiency and heat stress have been shown to alter root development by reducing primary root growth and reducing cell proliferation, respectively. Currently, identifying the dynamic temporal coordination of genetic responses to combined abiotic stresses remains a bottleneck. This is, in part, due to an inability to isolate specific intervals in developmental time where differential activity in plant stress responses plays a critical role. Here, we observed that iron deficiency, in combination with temporary heat stress, suppresses the expression of iron deficiency-responsive pPYE::LUC (POPEYE::luciferase) and pBTS::LUC (BRUTUS::luciferase) reporter genes. Moreover, root growth was suppressed less under combined iron deficiency and heat stress than under either single stress condition. To further explore the interaction between pathways, we also created a computer vision pipeline to extract, analyze, and compare high-dimensional dynamic spatial and temporal cellular data in response to heat and iron deficiency stress conditions at high temporal resolution. Specifically, we used fluorescence light sheet microscopy to image *Arabidopsis thaliana* roots expressing CYCB1;1:GFP, a marker for cell entry into mitosis, every 20 min for 24 h exposed to either iron sufficiency, iron deficiency, heat stress, or combined iron deficiency and heat stress. Our pipeline extracted spatiotemporal metrics from these time-course data. These metrics showed that the persistency and timing of CYCB1;1:GFP signal were uniquely different under combined iron deficiency and heat stress conditions versus the single stress conditions. These metrics also indicated that the spatiotemporal characteristics of the CYCB1;1:GFP signal under combined stress were more dissimilar to the control response than the response seen under iron deficiency for the majority of the 24-h experiment. Moreover, the combined stress response was less dissimilar to the control than the response seen under heat stress. This indicated that pathways activated when the plant is exposed to both iron deficiency and heat stress affected CYCB1;1:GFP spatiotemporal function antagonistically

## Introduction

Abiotic stresses, such as low iron bioavailability (iron deficiency, −Fe) or high ambient temperatures (heat stress, Heat), negatively impact key important plant processes, including growth, development, and reproduction. The effects of iron deficiency stress range from impaired chlorophyll biosynthesis and chloroplast maintenance in shoots to reduced cellular respiration and mitochondrial development in roots (Thimm et al., 2001; López-Bucio et al., 2003; Rout and Sahoo, 2015). Additionally, iron deficiency generally results in reduced primary root growth in favor of increased lateral root growth due to modulations in cell division within the primary and lateral root meristems (López-Bucio et al., 2003; Gharagozloo et al., 2008; Gruber et al., 2013; Hilo et al., 2017). Similarly, heat stress impairs photosynthesis, resulting in reduced biomass, reduced primary root growth, and arrested cell division within the root meristem (Larkindale et al., 2005; Wahid et al., 2007; Zhao et al., 2014). Overall, heat stress impairs many cellular processes via a cytotoxic accumulation of Reactive Oxygen Species (ROS), while also hindering enzyme and membrane function (Larkindale et al., 2005; Mangelsen et al., 2011; Tsukagoshi, 2012). Heat stress also increases the rate of cells transitioning into the elongation zone within the root meristem (Feraru et al., 2019). At its most severe, heat stress causes apoptosis (Larkindale et al., 2005). Moreover, heat stress has been implicated in several unrelated stress pathways, such as heavy metal and oxidative stresses (Larkindale et al., 2005; Kilian et al., 2007; Swindell et al., 2007; Hahn et al., 2013). While these stress response studies have shed light on many physiological and molecular effects, iron deficiency stress and heat stress responses have been traditionally studied in isolation. Given that, in field conditions, it is common for plants to experience stresses in combination rather than as isolated stress events (Thimm et al., 2001; Suzuki et al., 2014; Corrales et al., 2017; Carvalho et al., 2018), it is necessary to understand the existence of any interplay between stress response pathways when plants are exposed to multiple stresses. Experimental and computational tools that extract spatial and temporal similarities/differences in the molecular response of plants under both combinatorial and individual stresses would provide insight into the existence of interplay between two stress response pathways. This type of analysis requires quantifying how these stresses, both individually and combinatorially, contribute to the magnitude of each respective stress response with respect to a control condition. This analysis requires identifying the existence of an interaction between stress pathways. If there is interaction, it is also necessary to understand how the interaction influences function (antagonistically or agonistically).

While recent studies have indicated that there does not exist a universal stress regulator in plants, there is evidence for commonality in transcriptional regulation within groups of stress types, particularly in heat stress responses (Kilian et al., 2007; Swindell et al., 2007; Iyer-Pascuzzi et al., 2011; Kilian et al., 2012; Hahn et al., 2013; Zandalinas et al., 2018). A commonly observed phenomenon for the response of plants under stress is a change in cell cycle progression in actively proliferating regions such as the root meristem (Skirycz et al., 2011). Obtaining data that quantifies the time, location, and duration in which cell cycle progression is altered under single and combinatorial stress conditions could provide substantial insight into the interplay between iron deficiency and heat stress response pathways that are activated under combinatorial stress conditions. These data may also provide quantitative insight into how plants regulate overall cell proliferation; a key aspect of organ growth (Sakamoto et al., 2016).

In this work, we theorize that the comparison of spatiotemporal cell cycle patterns extracted from single and combinatorial stress data may allow us to 1) infer the temporal characteristics of stress specific pathways under combinatorial stress conditions, and 2) decipher whether stress specific pathways interact with one another and if that interaction functions antagonistically. We first quantified the dynamics of transcriptional luciferase fusions for two known −Fe response genes, POPEYE (PYE) and BRUTUS (BTS) in response to iron deficiency stress (−Fe), heat stress (Heat), and a combination of both stresses (−Fe+Heat). Since plants can be exposed to high temperature fluctuations over a 24-h period in field conditions, heat stress experimental regimes typically involve a short application of heat (60–90 mins) (Yeh et al., 2012; Silva-Correia et al., 2014). For this reason, we chose to implement heat stress by applying 38°C for 80 min. However, we applied iron deficiency stress for the full 24 h because this is a nutritional stress that plants are more commonly exposed to for days or longer. We observed that under the combinatorial −Fe+Heat stress condition, transcriptional activation of these genes is suppressed by Heat within the 24-h time frame, revealing antagonism between the output of these stress pathways with respect to these −Fe response genes. To gain further insight into how these combinatorial stress conditions affect molecular mechanisms associated with root development, we developed a computational approach to extract non-iron deficiency specific high-dimensional data from microscopy images obtained after exposing *Arabidopsis thaliana* seedlings expressing CYCB1;1:GFP (a proxy for cell entry into division) (Menges et al., 2005; de Luis Balaguer et al., 2016; Schnittger and De Veylder, 2018) to these conditions. We used Light Sheet Fluorescence Microscopy (LSFM), which offers low phototoxicity in fluorescent molecules, to acquire high temporal imaging data over 24-h time-course experiments (Reynaud et al., 2015; Ovečka et al., 2018). We developed the BioVision Tracker (BVT) image analysis pipeline to analyze root growth dynamics and track CYCB1;1:GFP expression within the meristematic region of the root over time. We used these tracking data to extract spatiotemporal metrics, which captured similarities and differences in spatiotemporal CYCB1;1:GFP expression patterns over fine intervals of time in response to −Fe, Heat, and −Fe+Heat. We show that our computational pipeline was capable of extracting useful spatiotemporal metrics from high throughput microscopy images, which revealed that Heat and −Fe responses interact with one another in an antagonistic manner. This technology facilitates the design of further studies with unprecedented specificity into how simultaneous plant stress responses function on a given output, which will potentially inform agricultural efforts in maintaining consistent crop growth despite impending climate and environmental changes.

## Results

### Iron Deficiency Responsive Genes Are Suppressed When Introduced to Both Iron Deficiency and Heat Shock

**Figure 1:**
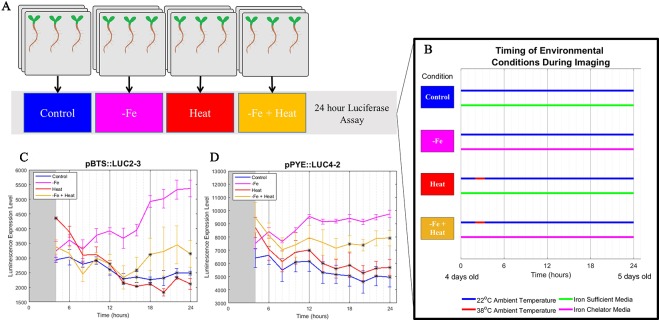
Bioluminescence assay on POPEYE and BRUTUS. **(A**, **B)** A 24-h bioluminescence assay was performed to measure the expression levels of two major iron deficiency response genes POPEYE (PYE) and BRUTUS (BTS). Two lines each of pPYE::LUC (pPYE::LUC5-5 and pPYE::LUC4-2) and pBTS::LUC (pBTS::LUC5-1 and pBTS::LUC2-3) seedlings were grown on plates for 4 days and then exposed to Control, −Fe, Heat, or −Fe+Heat conditions (three biological replicates containing three seedlings for each condition). **(C**, **D)** Imaging began at 4 h and was conducted every 2 h following. These luminescence expression signals show that the expression of BRUTUS and POPEYE were suppressed over time when the seedlings were introduced to −Fe+Heat in comparison to −Fe (*p < 0.05 in comparison to the −Fe condition using a two-sample t-test). Here, lines pBTS::LUC2-3 and pPYE::LUC4-2 are shown, whereas supplementary material shows all four lines. Error bars show the standard error across biological replicates.

To gain insight into if and how Heat stress affects the −Fe response under combinatorial stress, we examined the dynamics of transcriptional luciferase fusions for two known −Fe response genes, *POPEYE* and *BRUTUS* in *A. thaliana* seedlings. PYE acts as a regulator to many iron deficiency-specific genes while BTS is tightly coregulated with PYE such that both genes are transcriptionally induced in response to −Fe (Long et al., 2010). Two independent transgenic lines expressing pPOPEYE::luciferase (pPYE::LUC4-2 and pPYE::LUC5-5) or BRUTUS::luciferase (pBTS::LUC5-1 and pBTS::LUC2-3) were exposed to either 1) control media (Control); 2), −Fe media (−Fe), 3) control media and subjected to 80 min of heat stress (Heat); or 4) −Fe media and subjected to 80 min of heat stress (−Fe+Heat) (**Figures 1A, B**; see *Materials and Methods*). We performed a bioluminescence assay for our treatments to measure p*BTS::LUC* and p*PYE::LUC* expression levels in response to our variety of stress conditions. We observed that the expression levels of p*PYE::LUC* and p*BTS::LUC* were significantly higher for −Fe compared to the Control and Heat conditions within 12 h of the experiment (p < 0.05) (**Figures 1C, D** and **Supplementary Figure 2**). This was expected since *POPEYE* and *BRUTUS* are upregulated within 12 h of –Fe induction based on previous studies (Long et al., 2010). However, the expression levels were significantly higher under the –Fe than under the –Fe+Heat condition within 16 h for p*BTS::LUC* and within 18 h for p*PYE::LUC* (p < 0.05). This result was unexpected since BRUTUS and POPEYE are not known to be heat stress-responsive and it was not anticipated that exposure to −Fe+Heat would significantly disrupt the expression of either *BRUTUS* or *POPEYE*. These statistically different expression profiles suggest that there was interaction between the two pathways under a combination of stress. Furthermore, p*BTS::LUC* and p*PYE::LUC* expression levels appear to be suppressed when Heat is added in combination to −Fe which suggests the pathways are functioning antagonistically with one another. Therefore, we investigated further into the interplay of these two stress pathways by looking at root growth and molecular markers correlated with root growth and cell division that are not specific to either stress to determine if there exist similar antagonistic characteristics on the root itself.

### Imaging Pipeline and Root Growth Assay Shows Root Growth Rate is Higher in −Fe+Heat Than in −Fe or Heat Alone

**Figure 2:**
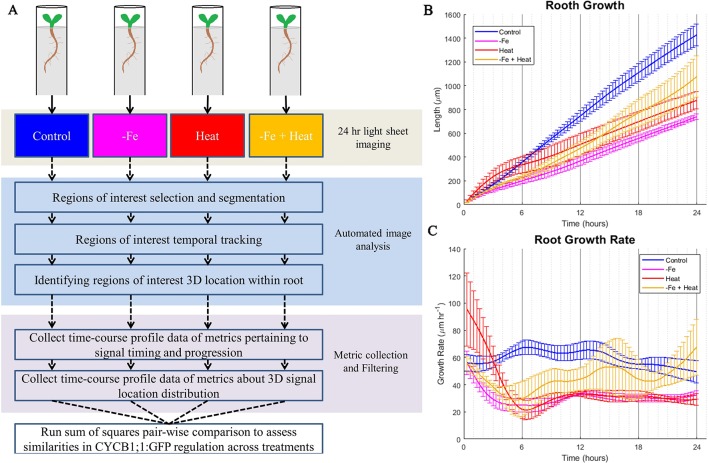
Overall computational pipeline and root growth assays. **(A)** The overall flow of our experimental setup and computational approach. *A. thaliana* plants were grown in FEP tubes for 4 days and then treated to 1 of 4 of the same environmental conditions as the luciferase assay for 24 h while being imaged within the ZEISS Lightsheet Z.1 every 20 min. CYCB1;1:GFP, a proxy for entry into cell division, was visualized through a fluorescent channel. The images were then processed to segment, track, and locate CYCB1;1:GFP fluorescent regions of interest within the 3D locations of the root. Then, metrics were derived from the automated image analysis data to give temporal profiles of spatiotemporal CYCB1;1 data from fluorescent signals. These profiles were used to quantify similarities of cell division regulation across stress conditions. The total root growth during the course of the experiment **(B)** and the root growth rate **(C)** was recorded every 20 min over the 24 experiment for n = 3 to 4 biological replicates.

To gain insight into these stress responses at the molecular and physiological level, we grew *A. thaliana* CYCB1;1:GFP seedlings in a MAGIC growth and imaging chamber (de Luis Balaguer et al., 2016) for 4 days and subsequently imaged them by a light sheet microscope every 20 min for 24 h under the same conditions as was used in the luciferase assay (**Figures 2A, B**). Since cell division is a contributor to root growth, second to root cell elongation, we chose CYCB1;1:GFP as a proxy for cell division because of its significant involvement in signaling mitosis (López-Bucio et al., 2003; Tsukagoshi, 2012). We chose to observe CYCB1;1:GFP every 20 min because CYCB1;1 expression, on average, has a duration of 3 h, which gave us about nine samples per occurrence (Yin et al., 2014). Using the digital images from both the fluorescent and brightfield channels of the light sheet microscope, we segmented, processed, and tracked CYCB1;1:GFP Regions of Interest (ROI) within the root with our custom automated image analysis software as described below in Development of an Automated Image Analysis Software to Extract Quantitative Spatiotemporal Metrics of CYCB1;1:GFP (adapted from Buckner et al., 2018). We utilized the tracking portion of our pipeline to assess root growth by quantifying the global movement of the root between sequential images. These data were collected every 20 min, aggregated into a time signal, and filtered using a low-pass filter to reduce noise and jitter (see *Materials and Methods*). As expected, the −Fe, Heat, and −Fe+Heat conditions resulted in stunted overall root growth within the scope of the 24-h experiment (**Figure 2B**). However, for the −Fe+Heat condition, the total root growth over the 24 h was more than the total root growth under either of the individual stress conditions (**Figure 2B**). By observing the root growth rate over time (**Figure 2C**) we were able to conclude that after 5 h of the experiment, the rate in which roots undergoing −Fe+Heat conditions were growing was faster than the roots undergoing −Fe or Heat conditions individually. This suggests that due to the response to both of these stresses, an interaction between pathways may have occurred to result in increased root growth rate. Although our assay does not indicate the specific molecular mechanism by which this interaction occurred, the interaction appear antagonistic since the suppression of root growth was decreased under the combinatorial condition.

To further assess the characteristics that this combination of stresses induced at the molecular level, we modified our existing computational pipeline (Buckner et al., 2018) to provide an automated process for collecting temporal characteristics of cells newly expressing CYCB1;1:GFP as well as the perdurance of CYCB1;1:GFP signal. It also collected spatial information of where in the root the CYCB1;1:GFP signal was detected. These CYCB1;1:GFP ROI tracking data were used to generate profiles of 10 spatiotemporal metrics (Material and Methods) over the 24-h experimental period. The temporal profiles of these 10 metrics were analyzed and compared numerically using a sum of squares approach to quantify similarities and differences between single and combinatorial stress responses (**Figure 2A**).

### Development of an Automated Image Analysis Software to Extract Quantitative Spatiotemporal Metrics of CYCB1;1:GFP

**Figure 3:**
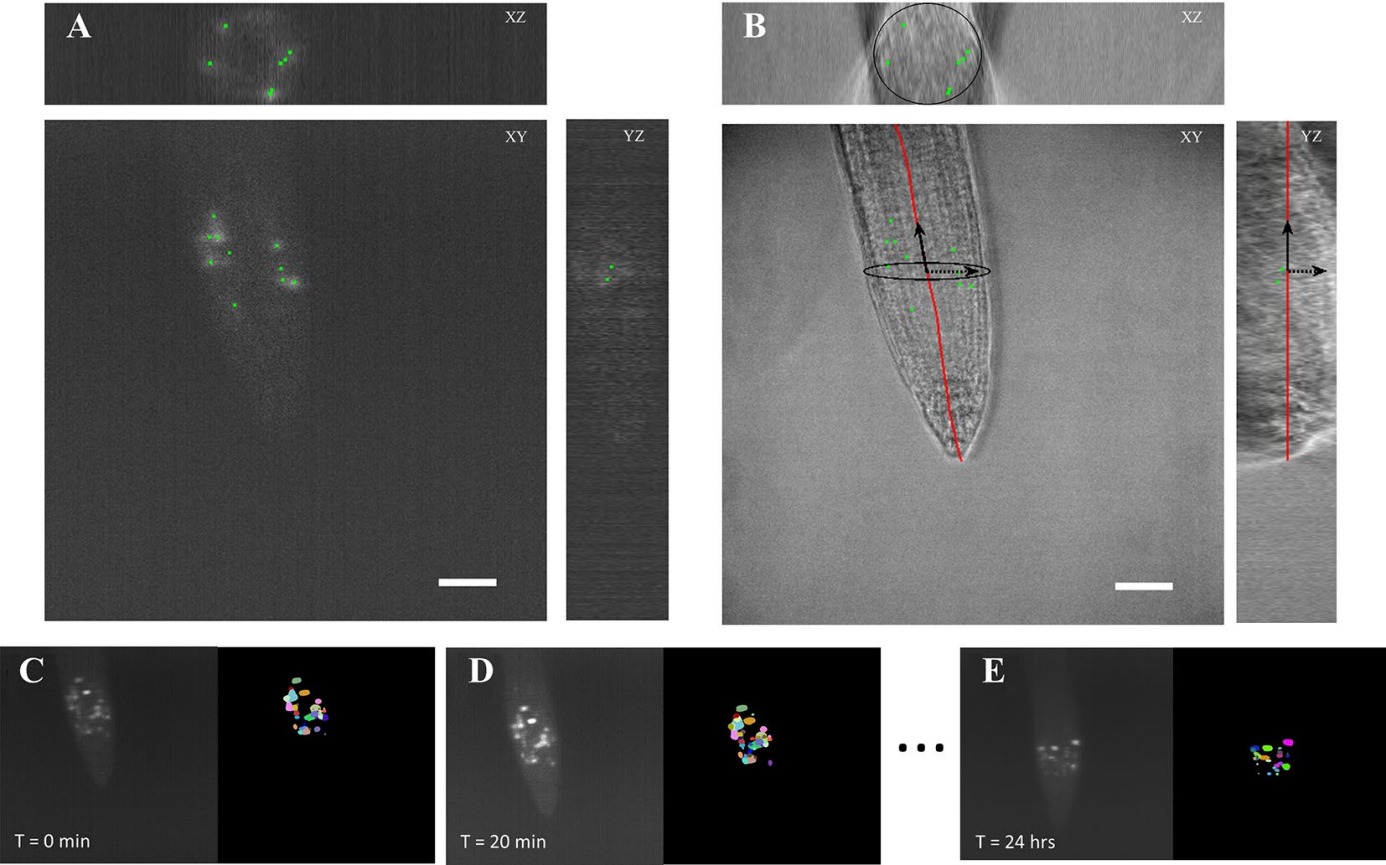
Automated image analysis on 3D light sheet microscopy images. **(A)** A 3D fluorescent image was taken every 20 min capturing ROIs of the CYCB1;1:GFP signal. The image analysis software distinguished the different ROIs as individual instances. **(B)** A corresponding 3D brightfield image was taken every 20 min in the light sheet growth chamber to capture the overall structure of the *A. thaliana* root. The ROI locations were on to the processed coordinate system of the root. Scale bars = 50 *μ*m. **(C**–**E)** Segmentation and tracking of the 3D images max projected onto 2D images. Segmented ROIs of the same color indicates the same region in different time stamps.

We developed the BioVision Tracker (BVT) image analysis software to track ROIs in the time course microscopy images using a method that we adapted from our previously developed algorithm (Buckner et al., 2018). BVT first processes 3D fluorescence microscopy images (**Figure 3A**) by selecting ROIs using an image intensity threshold and segmenting each ROI using a watershed algorithm for each image in the time-course (**Figures 3C–E**). BVT then uses image registration techniques to track the movement of the ROIs within the scope of the microscope’s field of view over time (**Figures 3C–E**). Additionally, BVT is able to define a root-specific coordinate system that localizes the ROIs within specific portions of the root by processing the 3D images from the Brightfield channel (**Figure 3B**). This is done by using an unsupervised clustering algorithm on the Brightfield image’s gradient data to estimate which voxels in the image contain root tissue and which do not (Buckner et al., 2018).

We processed the images taken by the light sheet using the BVT software to provide tracking information about ROIs that fluoresce the CYCB1;1:GFP signal in the meristematic region of the roots. We extracted two major categories of data from this software: 1) the location of each ROI with respect to the distance from the tip of the root and the longitudinal axis and 2) the tracking information about which ROIs continued to produce signal in sequentially sampled images and for how long (**Figure 3**). These data were aggregated for each environmental condition (Control, −Fe, Heat, and −Fe+Heat) and used to produce 10 spatiotemporal metrics that capture the characteristics of the CYCB1;1:GFP signal under single and combinatorial stress.

**Figure 4:**
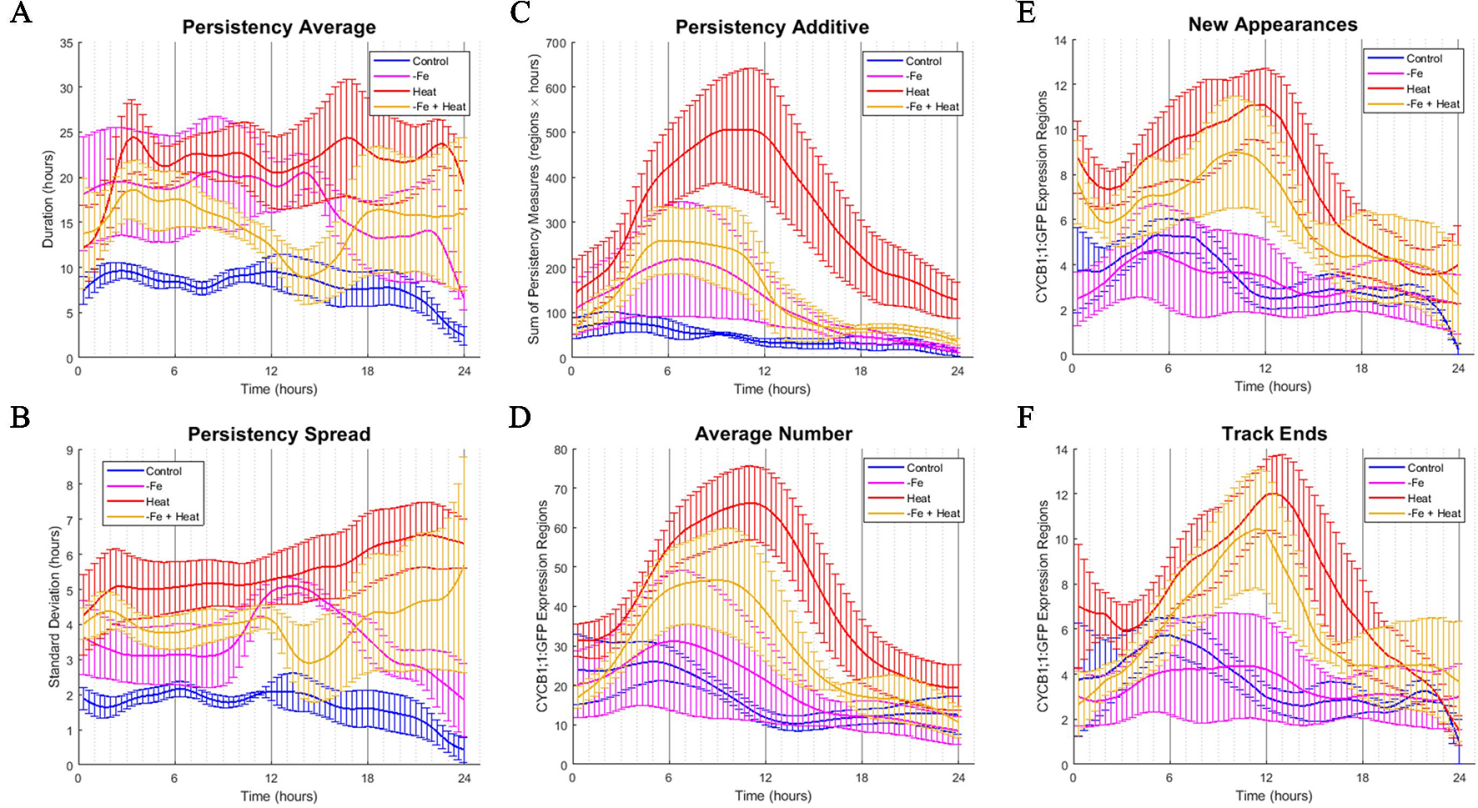
Time-course profiles of individual metrics obtained from the image analysis software. **(A**–**F)** Metrics obtained from image analysis of all conditions over a 24-h time period. Error bars indicate standard error from the biological replicates (n = 3 to 4). **(A)** PERSISTENCY AVERAGE metric showing the average time (in hours) that ROIs were tracked. This corresponds to how long cells were expressing CYCB1;1:GFP. **(B)** PERSISTENCY SPREAD metric showing the standard deviation of ROI persistency measures. This corresponds to the variety of lengths of time that cells expressed CYCB1;1:GFP. **(C)** PERSISTENT ADDITIVE metric showing the cumulative sum of persistency measures from detected ROIs at any one time. **(D)** AVERAGE NUMBER metric showing the average number of ROIs detected by the software at any one time. An increase in AVERAGE NUMBER corresponds to an increased number of cells producing CYCB1;1:GFP **(E)** NEW APPEARANCES metric showing the average number of newly appearing ROIs at any one time. This corresponds to the average number of new cells expressing CYCB1;1:GFP **(F)** TRACK END metric showing the average number of ROIs ceasing to be tracked by the software at any one time.

The 10 spatiotemporal metrics, derived from the BVT tracking data characterized the spatiotemporal CYCB1;1:GFP ROI dynamics for each condition in 20-min increments over the 24-h experimental period. **Table 1** gives the definition of 6 of the 10 metrics, along with the biological implications as it relates to CYCB1;1:GFP. The remaining four metrics are described in the *Materials and Methods*. **Figure 4** shows 6 of the 10 spatiotemporal metrics that were collected to measure the temporal dynamics of the CYCB1;1:GFP expression. The additional four metrics were collected to measure overall spatial distributions of CYCB1;1:GFP signal over time with respect to the distance from the tip and the longitudinal axis of the root and are shown in **Supplementary Figure 1**.

**Table 1:** Temporal metric descriptions.

Metric	Technical description	Biological description (CYCB1;1)
PERSISTENCY AVERAGE	Each ROI tracked from the software has a persistency value which is the amount of time (in hours) that ROI has been and will be tracked from the images. This metric is the average persistency measure of ROIs at a time point.	An increase in this metric suggests a longer sustained CYCB1;1 signal
PERSISTENCY SPREAD	The standard deviation of persistency measures of the ROIs at a time point.	An increase in this metric suggests that the duration of time of sustained CYCB1;1 signal in cells is highly variable.
PERSISTENT ADDITIVE	The cumulative sum of all ROI persistency measures at a time point.	An increase in this metric suggests higher overall CYCB1;1 production in the meristematic region
AVERAGE NUMBER	The number of ROIs detected by the software at a time point.	An increase in this metric suggests more individual cells are producing CYCB1;1
NEW APPEARANCES	The number of ROIs that first appear at a time point.	An increase in this metric suggests more cells are beginning to produce CYCB1;1
TRACK END	The number of ROIs that cease to be tracked at a time point	An increase in this metric suggests more cells are ceasing to produce CYCB1;1

The data from each of the 10 metrics were transformed into 10 time signals using a low pass digital filter across all conditions. The value of the time signal at a given time stamp was computed by averaging the data for the corresponding metric around that specific time (see *Materials and Methods*). This approach provided a way of reducing noise and jitter in the spatiotemporal metrics as sliding windows can behave much like a low pass filter. We first observed that the metrics describing spatial measurements did not show obvious distinctive differences between the temporal profiles of the different environmental conditions (**Supplementary Figure 1**). However, we found that CYCB1;1:GFP timing and progression were both greatly affected and highly dynamic under iron deficiency stress, heat stress, and a combination of both stresses when compared to the control over 24 h (**Figure 3**, **Table 1**).

To assess how single and combinatorial stresses altered CYCB1;1:GFP expression duration with respect to the control, we further examined the metrics associated with persistency (PERSISTENCY AVERAGE, PERSISTENCY SPREAD, and PERSISTENT ADDITIVE) and timing (AVERAGE NUMBER, NEW APPEARANCES, and TRACK END) in greater detail. We first examined metrics associated with persistency, or the amount of time (in hours) a uniquely identified ROI is tracked, as it relates to the average persistency across all detected ROIs at a given time stamp (PERSISTENCY AVERAGE), the variation around this average, (PERSISTENCY SPREAD), and additive sum of persistency of all detected ROIs (PERSISTENT ADDITIVE). These signals are shown in **Figures 4A–C**, respectively. We note that overall, under control conditions, these metrics remain constant with little variation. This suggests that the progression of cell cycle remains relatively constant for the portion of the cycle in which CYCB1;1:GFP is active over a 24-h period when no stress is applied. We also look at metrics associated with the timing of CYCB1;1:GFP characteristics, such as the average number of ROIs detected at a given time stamp (AVERAGE NUMBER), the number of new ROIs that appear at that time stamp (NEW APPEARANCES), and the number of ROIs that are no longer detectable at that time point (TRACK END). These signals are shown in **Figures 4D–F**, respectively. For the control, **Figures 4D–F** showed a maximum value around 6 h for these timing metrics, indicating that under control conditions regions currently expressing, starting to express, and ceasing to express CYCB1;1:GFP had peaked around this time. We compared the characteristics of these timing and persistency metrics under control conditions to the metrics obtained under −Fe, Heat, and −Fe+Heat conditions to assessing differences in magnitude and temporal characteristics.

We observed that the PERSISTENCY AVERAGE and PERSISTENCY SPREAD signals were consistently higher for all three stress conditions than for the control while also maintaining larger variation throughout most of the 24-h window (**Figures 4A, B**). These results suggest that there is an increase in the length of time uniquely identified ROIs are tracked under these stresses which could potentially imply a stall in cell cycle, specifically during the stage of mitosis. While the data suggests increased persistence for all three stress conditions, the profiles of these two metrics for the −Fe+Heat condition does not appear to strictly follow that of either single stress. Furthermore, it appears that the PERSISTENCY AVERAGE of the −Fe+Heat condition was lower than either single stress which may be associated with higher growth rates for this condition especially between 12 and 18 h in which −Fe+Heat and Control values of PERSISTENCY AVERAGE overlapped closely. During the same 12–18-h period, root growth rate (**Figure 2C**) under −Fe+Heat conditions accelerated to similar growth rates under Control conditions, which never occurred in either single stresses. Overall, this suggests a unique response in CYCB1;1:GFP persistency that correlates with altered root growth rate induced by combining −Fe and Heat.

The PERSISTENT ADDITIVE and AVERAGE NUMBER signals (**Figures 4C, D**) show similar increases in all three stress conditions in that all three contained a peak between 3 and 12 h. Thus, the data that our software collected was able to capture specific time points in which events associated with cell division spike. This spike could be explained by 1) the duration of time that cell division was being prolonged was increased for all stress conditions, 2) the rate in number of cells initiating division was larger than the rate of cells actually dividing, and/or 3) there was an increase of stress-induced DNA damage in cells since CYCB1;1 has also been shown to be produced in earlier stages of the cell cycle after DNA damage occurs is present in the cell (Schnittger and De Veylder, 2018). However, further experiments examining these time points would be needed to further conclude specifics about the effects of iron and heat stress on the cell cycle.

Finally, we examine the characteristics of the NEW APPEARANCES and TRACK END signals under stress conditions and compare them to the characteristics seen under the control. For the NEW APPEARANCES signal, we observed that for Heat, the increase of newly appearing ROIs was sustained and peaked around 12 h in contrast to both the control and −Fe conditions, which were sustained but peaked at less than 6 h. Finally, for the TRACK END signal, we observed delayed peaks for each stress condition compared to the control, where the peaks for each stress condition were located around 12 h.

Our computational approach allowed us to collect information that captured the dynamics of many spatiotemporal cell cycle characteristics in response to stress at an unprecedented resolution. Our method allowed us to extract many observations by expanding the data collected from image analysis into quantitative metrics. We observed that CYCB1;1:GFP was perdured in response to all three types of stress introduced here. We also found that many of the profiles for the −Fe+Heat condition had peaks and valleys at different times than either the −Fe or Heat conditions alone and, in most cases, the magnitude of response was different in −Fe+Heat than the profiles of either −Fe or Heat. This suggests that the time and the degree to which plants respond to −Fe+Heat with respect to CYCB1;1:GFP is distinct from that which occurs in response to −Fe or Heat individually. Therefore, we further investigated by aggregating all metrics collectively in a high-dimensional space to assess when, and to what degree, plants initiate specific stress responses, and how combining these stresses might affect these responses.

### Image Analysis Reveal That Characteristics of CYCB1;1:GFP in Combined Stress Are Different Than Single Stresses

**Figure 5:**
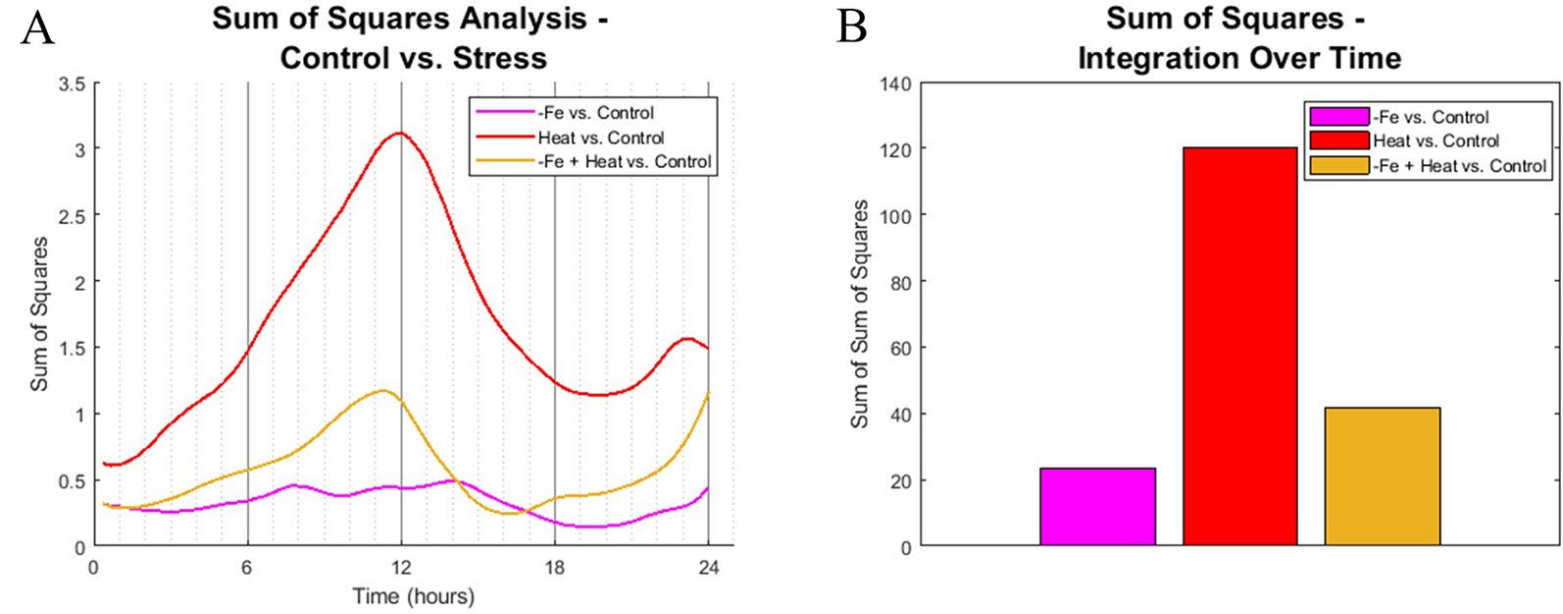
Sum of squares comparison of conditions. **(A)** Sum of squares analysis was performed every 20 min interval by comparing the high dimensional CYCB1;1:GFP data of stress-inducing experiments to the control. **(B)** The profiles from **(A)** were integrated across time to get overall dynamic similarities between treatments.

To identify similarities in spatiotemporal CYCB1;1:GFP behaviors between experimental conditions, we computed the sum of squared difference (SS) across all 10 metrics for all pairwise combination of stress vs. the control at each 20-min time point. Each metric was normalized between 0 and 1 so that no single metric would bear more weight than another. **Figure 5A** shows the SS values for each stress condition in comparison to the control. Note that a larger SS value corresponds to increased dissimilarity between the compared conditions.

We found that the spatiotemporal CYCB1;1:GFP data of the Heat and −Fe+Heat stress conditions diverged in behavior from the control early in the time course, which is indicated by increasing SS values for these curves within the first 3 h (**Figure 5A**), whereas the −Fe condition diverges very slightly from the control throughout the entire 24-h span. The SS values comparing the similarity between the Heat and Control conditions show distinctively different characteristics compared to the −Fe vs. Control profile in magnitude and timing, showing intervals of both increasing dissimilarity (0–12 h and 20–24 h, **Figure 5A**) and decreasing dissimilarity (12–20 h, **Figure 5A**). SS values for the −Fe+Heat vs. Control are different in magnitude from the aforementioned SS values, where the characteristics are more similar to the control than the Heat condition but less similar to the control than the −Fe experiment for most of the time course. This was further affirmed by looking at the integration of these SS signals over the entire 24-h experiment to assess overall dissimilarity (**Figure 5B**). This suggests that, according to our pipeline analysis, an interaction between stress pathways exists, but the overall effect on CYCB1;1:GFP expression is antagonistic.

## Discussion and Conclusion

Abiotic stresses affect development, in part, by altering the size, shape and number of cells, which is controlled by progression through the cell cycle. Our computational approach of using image analysis to quantify root growth showed that growth was inhibited more in −Fe and Heat conditions than in −Fe+Heat conditions. Furthermore, our approach was used to extract spatiotemporal gene expression data. This allowed us to observe many aspects of how and when CYCB1;1:GFP patterns are altered due to iron deficiency stress, heat stress, and a combination of both conditions, which have certain implications on cell division. We found that overall, CYCB1;1 spatiotemporal patterns were affected by both of these stresses individually and combinationatorially in as early as 3 h. However, the timing and magnitude to which they were affected appeared to differ under combined stress condition compared to single stress conditions alone. Our observation of CYCB1;1:GFP tracking revealed there to be an apparent temporal relationship between the temporal prolonging of CYCB1;1:GFP expression and root growth inhibition which could explain the increased root growth in the −Fe+Heat condition since the combined stress condition had overall lower PERSISTENCY AVERAGE; however, root growth is a physiological outcome that is controlled by a network of genes, and by assessing the characteristics of CYCB1;1:GFP, one may not be able to conclude causation of root growth, particularly because root cell elongation had likely been affected by either stress, which would also contribute to root elongation. Thus, we show how our approach has the potential to reveal underlying mechanisms by observing molecular and organ level characteristics at high temporal resolution. We conclude from these observations that these two stress pathways could interact which may cause the plant to regulate genes in a completely unique way apart from iron deficiency or heat stresses alone resulting in an antagonistic outcome on the biological reporters we have selected.

Our results are consistent with previous studies that have concluded not only that plant gene regulation cannot be predicted in many combinatorial stresses just by observing the regulation of each stress response individually, but also that combinatorial stresses trigger unique stress responses relative to that of single stress responses (Kilian et al., 2012; Rivero et al., 2014). Moreover, with respect to iron-centric studies, increased iron availability is implicated in promoting both root meristematic cell division and *CYCB1* expression in petunia and has established roles in regulating primary root elongation (Landsberg, 1996; López-Bucio et al., 2003; Gharagozloo et al., 2008; Gruber et al., 2013; Hilo et al., 2017). Furthermore, heat stress is also known to reduce primary root growth and promote G2/M phase arrest in root meristematic cells (Zhao et al., 2014). Established relationships with either iron or heat stress within roots are consistent with our findings of apparent cell cycle arrest, based on dynamics changes in CYCB1;1:GFP, in all stress conditions. Little is known about the combinatorial interactions of heat and iron stress in plants, particularly in root cells. However, it has been shown that moderate heat stress causes ferroptosis-like cell death in root hair cells. Ferroptosis, identified first in animal cells, is a type of cell death that occurs in response to moderate heat stress and is dependent on cellular iron availability and mediated by ROS accumulation (Mushegian, 2017). In recent studies, ferroptosis-like cell death has been identified in root hair cells in which moderate heat stress induces cell death that is mediated by ROS accumulation, as in animal cells (Distéfano et al., 2017). However, in combination with iron chelators, heat stress is unable to induce cell death; thus, iron deficiency via chelation has been shown to attenuate a moderate heat stress response (Distéfano et al., 2017). Similarly, our results suggest that heat stress and iron deficiency, which was induced by iron chelation, interact in an opposing manner to regulate the cellular life cycle. Moreover, heat stress triggers ROS production, resulting in either root acclimation to stress or cell death; however, heat stress in combination with other abiotic stresses, such as drought or salinity, results in unique patterns of ROS production relative to those produced by heat single stress (Kilian et al., 2012; Choudhury et al., 2017). It is likely that heat stress responses, in concert with iron deficiency stress responses function antagonistically to each other so that cellular ROS production is inhibited and, thus, ROS-induced or iron-dependent (ferroptosis) cell death was mitigated. Moreover, it is also possible that the stress responses induced by iron deficiency primed, or acclimated, the root to the subsequent heat stress condition, especially since priming, or desensitization of a plant from one stress by another previous stress has been observed (Kilian et al., 2012).

Since iron deficiency causes the malnutrition of billions of individuals worldwide and there is currently a rise in global temperatures, it is very likely that these two stresses commonly appear in combination to many crops all over the world (Stein, 2010). While our laboratory growth conditions may not fully reflect field conditions in which crops are grown, we attempted to emulate aspects of field-like characteristics such as a short (80 min) heat stress application and constant 24-h iron deficiency application, which is commonly used to reflect fluctuating heat over a 24-h period and prolong nutrient deficiencies, respectively. Our findings and methods shed new light on how plant responses to combinatorial stress conditions may be non-intuitive. Moreover, we provide a computational approach that can be adapted to study a broad application of spatiotemporal dynamics of gene regulation under diverse developmental and environmental conditions. We have designed our approach such that it can be customizable to many different applications for gaining insight on the effects that environment has on plant growth. Specifically, our approach can be used to transform high volumes of fluorescence microscopy image data, containing specimens with fluorescent-tagged genetic markers, into quantitative spatiotemporal metrics. These metrics can then, as shown here, give biological insight into the timing and characteristics of plant responses under single and multiple stresses.

## Materials and Methods

### Plant Growth and Seed Preparation

The *Arabidopsis thaliana* pCYCLINB1;1:CYCLINB1;1::GFP in a Columbia (Col-0) background was used for all experiments. Seeds were sterilized in 70% ethanol for 5 min followed by incubation in 30% bleach and 0.02% Triton X-100 for 15 min. Then, seeds were rinsed 3 times in sterile water and stratified at 4°C for at least 2 days. Seeds were germinated and grown in a MAGIC 3D printed growth chamber as described in (de Luis Balaguer et al., 2016). Each seed was germinated in 13-mm-long FEP tube, containing iron-sufficient (+Fe) MS low gelling media, consisting of standard Murashige and Skoog medium with 0.05% MES (w/v), 1% (w/v) sucrose, 0.4% (w/v) low gelling agar, and 0.1-mM FeEDTA substituted for iron sulfate. Each tube was oriented vertically, held upright by standard +Fe MS solid media in plates which were kept at 22°C under a 16-h-light and an 8-h-dark period in environmentally-controlled plant growth chambers (Percival Scientific).

On the 4th day after planting, seedlings were prepared for imaging. To induce iron deficiency, +Fe low gelling medium was replaced with −Fe low gelling medium, which had the same composition of +MS low gelling media, except that 300 µM ferrozine was added as an iron chelator. For the biological replicates that were induced with heat stress, the imaging chamber was programmed to incubate the specimens at 38°C for 80 min starting at the 2-h mark from the beginning of the imaging experiment. For all other times outside of the heat stress window and for specimens not induced with heat stress, the chamber was programmed to incubate at 22°C. Each experiment, iron sufficient (+Fe), iron deficient (−Fe), heat stress (Heat), and iron deficient with heat stress (−Fe+Heat), used 3–4 seedlings as individual replicates.

### Light Sheet Microscopy and Imaging Chamber

The ZEISS Lightsheet Z.1 microscope (Carl Zeiss, Germany) was used for all imaging experiments. All settings related to imaging configuration and imaging chamber environment of the microscope were adjusted using the ZEN software from ZEISS.

The MAGIC chamber was lowered into the light sheet where the meristematic region of each root was imaged using a W Plan-Apochromat 20x/1.0 NA objective (Carl Zeiss, Germany). Two image channels were taken simultaneously of each plant, a 3D fluorescent channel and a 3D brightfield channel. In the fluorescent channel, the laser was set to single-side excitation with settings of 488 nm, 50 mW, laser intensity set to 60%, and the exposure time was set to 29.97 ms. The SBS LP 560 beam splitter and BP505-545 emission filter were used to detect GFP emissions. Both the fluorescent images and the brightfield images were taken at a pixel resolution of 0.23 µm × 0.23 µm and a z-slice interval of 3.33 µm. The microscope was programmed to image each root every 20 min for 24 h. Every 20 min from the beginning to the end of any experiment is considered a timestamp. This time interval was chosen because it was determined to be a good sampling frequency for observing cell cycle changes in *A. thaliana*.

### Dynamic Cell Cycle Metrics Descriptions and Low Pass Filtering

The images taken from the light sheet were processed to track fluorescent CYCB1;1:GFP ROI over space and time using the BioVision Tracker software (Buckner et al., 2018). We further processed the data about the ROIs that were collected from the software into metrics that help characterize average spatiotemporal CYCB1;1:GFP signal patterns at any one time stamp. The following metrics were collected for each image that was taken.

1. PERSISTENCY AVERAGE (*X*_1_)—The average persistency measure of all ROIs detected.
2. PERSISTENCY SPREAD (*X*_2_)—This is the standard deviation of collected persistency measures from all ROIs in a single time point.
3. PERSISTENCY ADDITIVE (*X*_3_)—Each ROI has a persistency measure which is the length of time that each ROI has been and will be tracked (in hours). Persistency additive is the sum of all visible ROI’s persistency measures in a single time point.
4. AVERAGE NUMBER (*X*_4_)—This is the average number of ROIs detected at any one time stamp.
5. NEW APPEARANCE (*X*_5_)—This is the number of ROIs that first appear and begin to be tracked in the evaluated time stamp.
6. TRACK END (*X*_6_)—This is the number of ROIs that stopped being visible during that time stamp and thus stopped being tracked at that time.
7. TIP DISTANCE AVERAGE (*X*_7_)—This is the average distance away from the tip (in microns) ROIs appeared within the root at that time stamp.
8. TIP DISTANCE SPREAD (*X*_8_)—This is the standard deviation of ROI distances away from the tip of the root at that time stamp.
9. CENTER DISTANCE AVERAGE (*X*_9_)—This is the average distance away from the longitudinal axis (in microns) ROIs appeared within the root at that time stamp.
10. CENTER DISTANCE SPREAD (*X*_10_)—This is the standard deviation of ROI distances away from the longitudinal axis of the root at that time stamp.

These 10 metrics were collected for each 20-min imaging timestamp across all replicates in each environmental condition. Let $X_{i,k}^{r}(n)$,represent the discrete time signal for metric *k*, condition *i*, replicate *r*, and time stamp *t*, and let *h*(*n*) represent the following digital filter.

$$h\;(n)=\sum_{k=\;-\;4}^{S}\frac{1}{10}\delta (n-k),$$

where *δ* is the Dirac delta function.

All time signals were filtered by convolving them with *h*(*n*).

$$F_{i,k}^{r}(n)=X_{i,k}^{r}(n)*h(n)$$

The overall metric profiles were calculated using the average filtered signal across all replicates in each environmental condition. Here, *R* *_i_* is the number of biological replicates used for each condition *i*.

$$p_{i,k}(n)=\frac{1}{R_{i}}\sum_{r=1}^{R_{i}}F_{i,k}^{r}(n)$$

### Sum of Squares Pairwise Comparisons

Each metric profile was aggregated together to create a 10-dimensional profile. Here, *T* stands for the transpose operation of a matrix.

$$P_{i}(n)={\left[p_{i,1}{\left(n\right)}^{T},p_{i,2}{\left(n\right)}^{T},\ldots ,p_{i,10}{\left(n\right)}^{T}\right]}^{T}$$

To compare any two profiles (*P* *_i_*(*n*) vs. *P* *_j_*(*n*)), first each metric was normalized between 0 and 1.

$$P_{i}{}^{\prime }(n)=M^{-1}\times P_{i}(n),$$

where *M* is a 10 × 10-diagonal matrix that contains the highest observed value across all experiments for each of the 10 metrics.

A sum of squares (SS) operation was then completed across all metrics.

$$\underline{SS_{i,j}}(n)=\underline{1_{1\times 10}}\times ((P_{1}{}^{\prime }(n)-P_{j}{}^{\prime }(n))\circ (P_{i}{}^{\prime }(n)-P_{j}{}^{\prime }(n)))$$

To evaluate the overall SS value across all time (SST), the SS values for each time stamp were summed together. Here, *N* is the number of total time stamps found in the imaging experiment, which is 72 for this study.

$$SST_{i,j}=\underline{SS_{i,j}}(n)\times \underline{1_{N\times 1}}$$

### MAGIC Root Growth Assay

Using the 24-h time-course image data from the environmental experiments, a growth vector was calculated to represent the growth of the root between any two consecutive time stamps using image registration in the BioVision Tracker Software. The magnitude of each growth vector $g_{i}^{r}(n)$ was determined using a euclidean distance calculation for each replicate *r*, condition *i*, and time stamp *n*. This magnitude was translated into distances in microns from distances in voxels using the voxel resolution value (*α*) obtained from the light sheet metadata. For each root, the total growth at time stamp *n* was calculated using the following equation.

$$G_{i}^{r}(n)=\sum_{\mu =1}^{n}ag_{i}^{r}(\mu )$$

### Bioluminescence Assay

The promoter sequences of BTS: (3,000 bp) using 5’-caccATGAGATGAAATGTCTTATCTTTAT-3’ and 5’-TTCCCCCAAAGCTTATCTCCGTTTT -3’, and PYE: (1,120 bp) 5’-caccACCGCAAAACTATATATAGTATTT-3’ and 5’-CTTTGCTTTTATTACAGAACAAGA-3’, were amplified from genomic DNA from Columbia (Col-0) as the template. Each promoter region was cloned into pENTR/D-TOPO then transferred to the pFLASH vector, containing the firefly luciferase gene, containing a spectinomycin resistance gene. Transformation and selection proceeded as described in Long et al., 2010. The resultant reporter lines *pBTS::LUC5-1*, *pBTS::LUC2-3*, *pPYE::LUC4-2,* and *pPYE::LUC5-5* were germinated on iron sufficient MS media for 4 days. On the 4th day, seedlings were transferred to new (iron sufficient or iron deficient) media plates. Seedlings were sprayed with 5-mM D-luciferin (Goldbio) in 0.1% Triton X-100 8 h prior to transferring. After transfer, plates were acclimated in the percival for 2 h then half of the plates were put in a 38°C water bath for 80 min (as described above). Bioluminescence imaging was performed and the first image was taken 4 h after transfer. Images were acquired every 2 h with exposure times of 20 min across 2 consecutive days. Images were processed using software ImageJ (Schneider et al., 2012). All experimental treatments contain three biological replicates (n = 3) with three seedlings for each replicate. A two-sample t-test was run for all pairwise comparisons using the MATLAB function ttest2.m.

## Data Availability Statement

The datasets generated for this study are available on request to the corresponding author.

## Author Contributions

EB and IM collected data, analyzed the data, and wrote the manuscript. HC, AM, and CM collected data. RS, CW, and TL provided expert guidance and helped in writing the manuscript.

## Funding

EB was supported by the GAANN Fellowship in Molecular Biotechnology (grant #P200A160061). IM was supported by a fellowship from the Southern Regional Education Board (SREB) Doctoral Scholars Program. AM was supported by the National Science Foundation (grant 1120937, grant 1252376, and grant 1247427). Support to RS was provided by the National Science Foundation (NSF) (CAREER MCB 1453130). RS and TL were funded bilaterally by the NSF and the Biotechnology and Biological Sciences Research Council (BBSRC) (NSF MCB-1517058). TL, CW, and HC were supported by NSF MCB-1247427. TL is also funded by the USDA National Institute of Food and Agriculture, Hatch Project (Accession Number 101090). HC is also supported by a North Carolina Agriculture and Life Sciences Research Foundation’s Innovation Award.

## Conflict of Interest

The authors declare that the research was conducted in the absence of any commercial or financial relationships that could be construed as a potential conflict of interest.

## References

[B1] Buckner E. Ottley C. Williams C. de Luis Balaguer A. Melvin C. E. Rosangela Sozzani R. (2018). "Tracking gene expression via light sheet microscopy and computer vision in living organisms". 2018 40th Annual International Conference of the IEEE Engineering in Medicine and Biology Society (EMBC) (IEEE), Honolulu, HI 818–821. 10.1109/EMBC.2018.8512416 30440517

[B2] Carvalho A. Leal F. Matos M. Jse Lima-Brito J. (2018). "Effects of heat stress in the leaf mitotic cell cycle and chromosomes of four wine-producing grapevine varieties". Protoplasma 255 (6), 1725–1740. 10.1007/s00709-018-1267-4 29789939

[B3] Choudhury F. K. Rivero R. M. Blumwald E. Mittler R. (2017). Reactive oxygen species, abiotic stress and stress combination. Plant J. 90 (5), 856–867. 10.1111/tpj.13299 27801967

[B4] Corrales A. R. Carrillo L. Lasierra P. Nebauer S. G. Dominguez-Figueroa J. Renau-Morata B. (2017). "Multifaceted role of cycling DOF factor 3 (CDF3) in the regulation of flowering time and abiotic stress responses in *Arabidopsis*". Plant Cell Environ. 40 (5), 748–764. 10.1111/pce.12894 28044345

[B5] de Luis Balaguer M. A. Ramos-Pezzotti M. Rahhal M. B. Melvin C. E. Johannes E. Horn T. J. (2016). "Multi-sample arabidopsis growth and imaging chamber (MAGIC) for long term imaging in the ZEISS lightsheet Z.1". Dev. Biol. 419 (1), 19–25. 10.1016/j.ydbio.2016.05.029 27235815

[B6] Distéfano A. Martin M. Córdoba J. Bellido A. D'Ippólito S. Colman S. (2017). “Heat stress induces ferroptosis-like cell death in plants”. J. Cell Biol. 216 (2), 463–476. 10.1083/jcb.201605110 28100685 PMC5294777

[B7] Feraru E. Feraru M. I. Barbez E. Waidmann S. Sun L. Gaidora A. Kleine-Vehn J. (2019). “PILS6 is a temperature-sensitive regulator of nuclear auxin input and organ growth in *Arabidopsis thaliana*”. Proc. Natl. Acad. Sci. 116(9), pp.3893-3898. 10.1073/pnas.1814015116 30755525 PMC6397578

[B8] Gharagozloo M. Khoshdel Z. Amirghofran Z. Zahra Khoshdel Zahra Amirghofran(2008). “The effect of an iron (III) chelator, silybin, on the proliferation and cell cycle of jurkat cells: A comparison with desferrioxamine.” Eur. J. Pharmacol. 589(1–3): 1–7. 10.1016/j.ejphar.2008.03.059 18619590

[B9] Gruber B. D. Giehl R. F. H. Friedel S. von Wirén N. (2013). "Plasticity of the Arabidopsis root system under nutrient deficiencies". Plant Physiol. 163 (1), 161–179. 10.1104/pp.113.218453 23852440 PMC3762638

[B10] Hahn A. Kilian J. Mohrholz A. Ladwig F. Peschke F. Dautel R. (2013). Plant core environmental stress response genes are systemically coordinated during abiotic stresses. Int. J. Mol. Sci. 14 (4), 7617–7641. 10.3390/ijms14047617 23567274 PMC3645707

[B11] Hilo A. Shahinnia F. Druege U. Franken P. Melzer M. Rutten T. (2017). “A specific role of iron in promoting meristematic cell division during adventitious root formation”. J. Exp. Bot. 68 (15), 4233–4247. 10.1093/jxb/erx248 28922771 PMC5853222

[B12] Iyer-Pascuzzi A. S. Jackson T. Cui H. Petricka J. J. Busch W. Tsukagoshi H. (2011). "Cell identity regulators link development and stress responses in the *Arabidopsis*root". Dev. Cell 21 (4), 770–782. 10.1016/j.devcel.2011.09.009 22014526 PMC3204215

[B13] Kilian J. Whitehead D. Horak J. Wanke D. Weinl S. Batistic O. (2007). “The AtGenExpress global stress expression data set: protocols, evaluation and model data analysis of UV-B light, drought and cold stress responses”. Plant J. 50 (2), 347–363. 10.1111/j.1365-313X.2007.03052.x 17376166

[B14] Kilian J. Peschke F. Berendzen K. W. Harter K. Wanke D. (2012). Prerequisites, performance and profits of transcriptional profiling the abiotic stress response. BBA - Gene Regul. Mech. 1819 (2), 166–175. 10.1016/j.bbagrm.2011.09.005 22001611

[B15] López-Bucio J. Cruz-Ramı´rez A. Herrera-Estrella L. (2003). "The role of nutrient availability in regulating root architecture". Curr. Opin. In Plant Biol. 6 (3), 280–287. 10.1016/S1369-5266(03)00035-9 12753979

[B16] Landsberg E. (1996). Hormonal regulation of iron-stress response in sunflower roots: A morphological and cytological investigation. Protoplasma 194 (1–2), 69–80. 10.1007/BF01273169

[B17] Larkindale J. Hall J. D. Knight M. R. Vierling E. (2005). “Heat stress phenotypes of *Arabidopsis* mutants implicate multiple signaling pathways in the acquisition of thermotolerance”. Plant Physiol. 138 (2), 882–897. 10.1104/pp.105.062257 15923322 PMC1150405

[B18] Long T. A. Tsukagoshi H. Busch W. Lahner B. Salt D. E. Benfey P. N. (2010). “The bHLH transcription factor POPEYE regulates response to iron deficiency in Arabidopsis roots.” The Plant Cell, 22 (7), 2219–2236. 10.1105/tpc.110.074096 20675571 PMC2929094

[B19] Mangelsen E. Kilian J. Harter K. Jansson C. Wanke D. Sundberg E. (2011). Transcriptome analysis of high-temperature stress in developing barley caryopses: early stress responses and effects on storage compound biosynthesis. Mol. Plant 4 (1), 97–115. 10.1093/mp/ssq058 20924027

[B20] Menges M. De Jager S. M. Gruissem W. Murray J. A. H. (2005). "Global analysis of the core cell cycle regulators of *Arabidopsis* identifies novel genes, reveals multiple and highly specific profiles of expression and provides a coherent model for plant cell cycle control". Plant J. 41 (4), 546–566. 10.1111/j.1365-313X.2004.02319 15686519

[B21] Mushegian A. A. (2017). Ferroptosis-like cell death in plants. Sci. Signaling 10 (468). 10.1126/scisignal.aan0450. 10.1126/scisignal.aan0450 28246195

[B22] Ovečka M. von Wangenheim D. Tomančák P. Šamajová O. Komis G. Šamaj J. (2018). "Multiscale imaging of plant development by light-sheet fluorescence microscopy". Nat. Plants 4 (9), 639–650. 10.1038/s41477-018-0238-2 30185982

[B23] Reynaud E. G. Peychl J. Huisken J. Tomancak P. (2015). "Guide to light-sheet microscopy for adventurous biologists". Nat. Methods 12 (1), 30–34. 10.1038/nmeth.3222 25549268

[B24] Rivero R. M. Mestre T. C. Mittler R. O. N. Rubio F. Gargia-Sanchez F. Martinez V. (2014). “The combined effect of salinity and heat reveals a specific physiological, biochemical and molecular response in tomato plants”. Plant Cell Environ. 37 (5), 1059–1073. 10.1111/pce.12199 24028172

[B25] Rout G. R. Sahoo S. (2015). "Role of iron in plant growth and metabolism". Rev. In Agric. Sci. 3, 1–24. 10.7831/ras.3.1

[B26] Sakamoto T. Sakamoto Y. Matsunaga S. (2016). Cell Division and Cell Growth. In Molecular Cell Biology of the Growth and Differentiation of Plant Cells (pp. 86–98). Boca Raton, FL: CRC Press, Taylor & Francis Group. 10.1201/b2031

[B27] Schneider C. A. Rasband W. S. Eliceiri K. W. (2012). "NIH image to ImageJ: 25 years of image analysis". Nat. Methods 9 (7), 671–675. 10.1038/nmeth.2089 22930834 PMC5554542

[B28] Schnittger A. De Veylder L. (2018). “The dual face of cyclin B1”. Trends In Plant Sci. 23 (6), 475–478. 10.1016/j.tplants.2018.03.015 29680634

[B29] Silva-Correia J. Freitas S. Tavares R. M. Lino-Neto T. Azevedo H. (2014). Phenotypic analysis of the Arabidopsis heat stress response during germination and early seedling development. Plant Methods 10, 7 (2014) 10.1186/1746-4811-10-7 24606772 PMC3975293

[B30] Skirycz A. Claeys H. De Bodt S. Oikawa A. Shinoda S. Andriankaja M. (2011). "Pause-and-stop: the effects of osmotic stress on cell proliferation during early leaf development in *Arabidopsis* and a Role for ethylene signaling in cell cycle arrest". Plant Cell 23 (5), 1876–1888. 10.1105/tpc.111.084160 21558544 PMC3123952

[B31] Stein A. J. (2010). “Global impacts of human mineral malnutrition”. Plant Soil 335 (1–2), 133–154. 10.1007/s11104-009-0228-2

[B32] Suzuki N. Rivero R. M. Shulaev V. Blumwald E. Mittler R. (2014). "Abiotic and biotic stress combinations". New Phytol. 203 (1), 32–43. 10.1111/nph.12797 24720847

[B33] Swindell W. R. Huebner M. Weber A. P. (2007). “Transcriptional profiling of *Arabidopsis* heat shock proteins and transcription factors reveals extensive overlap between heat and non-heat stress response pathways”. BMC Genomics 8 (1), 125. 10.1186/1471-2164-8-125 17519032 PMC1887538

[B34] Thimm O. Essigmann B. Kloska S. Altmann T. Buckhout T. J. (2001). “Response of *Arabidopsis* to iron deficiency stress as revealed by microarray analysis”. Plant Physiol. 127 (3), 1030–1043. 10.1104/pp.010191 11706184 PMC129273

[B35] Tsukagoshi H. (2012). “Defective root growth triggered by oxidative stress is controlled through the expression of cell cycle-related genes”. Plant Sci. 197, 30–39. 10.1016/j.plantsci.2012.08.011 23116669

[B36] Wahid A. Gelani S. Ashraf M. Foolad M. R. (2007). “Heat tolerance in plants: An overview.” Environ. Exp. Bot. 61 (3), 199–223. 10.1016/j.envexpbot.2007.05.011

[B37] Yeh C. H. Kaplinsky N. J. Hu C. Charng Y. Y. (2012). Some like it hot, some like it warm: phenotyping to explore thermotolerance diversity. Plant Sci. 195, 10–23. 10.1016/j.plantsci.2012.06.004 22920995 PMC3430125

[B38] Yin K. Ueda M. Takagi H. Kajihara T. Sugamata Aki S. Nobusawa T. (2014). “A dual-color marker system for in vivo visualization of cell cycle progression in *Arabidopsis*”. Plant J. 80 (3), 541–552. 10.1111/tpj.12652 25158977

[B39] Zandalinas S. I. Mittler R. Balfagón D. Arbona V. Gómez-Cadenas A. (2018). “Plant adaptations to the combination of drought and high temperatures”. Physiol. Plant. 162 (1), 2–12. 10.1111/ppl.12540 28042678

[B40] Zhao L. Wang P. Hou H. Zhang H. Wang Y. Yan S. (2014). "Transcriptional regulation of cell cycle genes in response to abiotic stresses correlates with dynamic changes in histone modifications in maize". PloS One 9 (8), e106070. 10.1371/journal.pone.0106070 25171199 PMC4149478

