# Supplementary material for: Automated Imaging, Tracking, and Analytics Pipeline for Differentiating Environmental Effects on Root Meristematic Cell Division
Source: Front Plant Sci. 2019 Nov 19;10:1487. doi: 10.3389/fpls.2019.01487 (PMC6877711; doi:10.3389/fpls.2019.01487)
Supplement: Supplementary file 1 [file Table_1.docx]

***Supplementary Material***

**1 Supplementary Code**

GitHub repository URL for the Matlab based BVT software: <https://github.com/edbuckne/BioVision_Tracker>

**2 Supplementary Figures and Tables**

**2.1 Supplementary Figures**


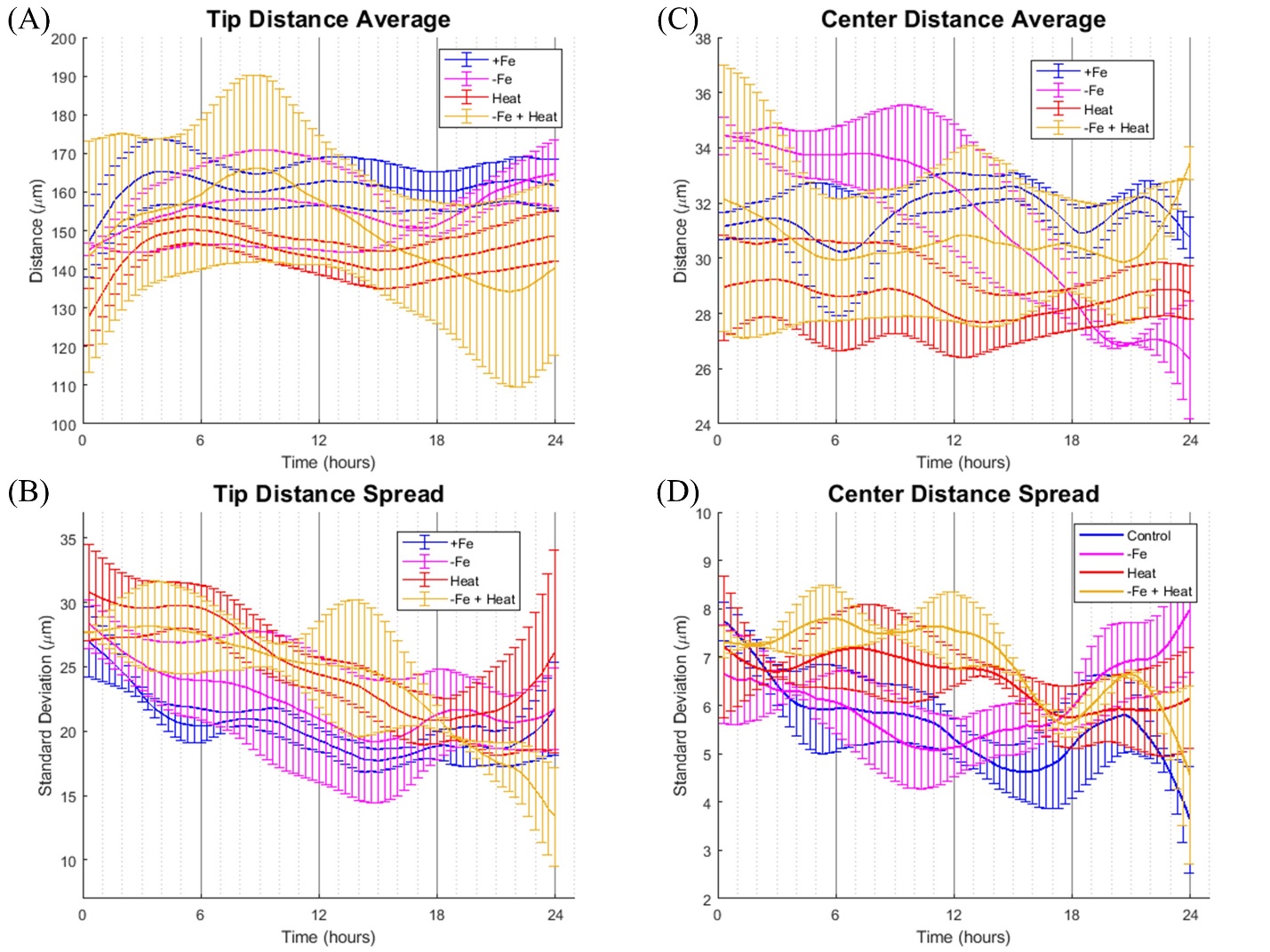


**Supplementary Figure 1.** Spatial distribution profiles of the 4 treatments. Error bars show the standard error of n = 3 to 4 biological replicates. (A) Tip Distance Average plots the distance away from the tip of the root the ROIs were detected on average over time. (B) Tip Distance Spread plots the standard deviation of ROI distances from the tip over time. (C) Center Distance Average plots the distance away from the center longitudinal axis of the root the ROIs were detected on average over time. (D) Center Distance Spread plots the standard deviation of ROI distances from the center longitudinal axis over time.


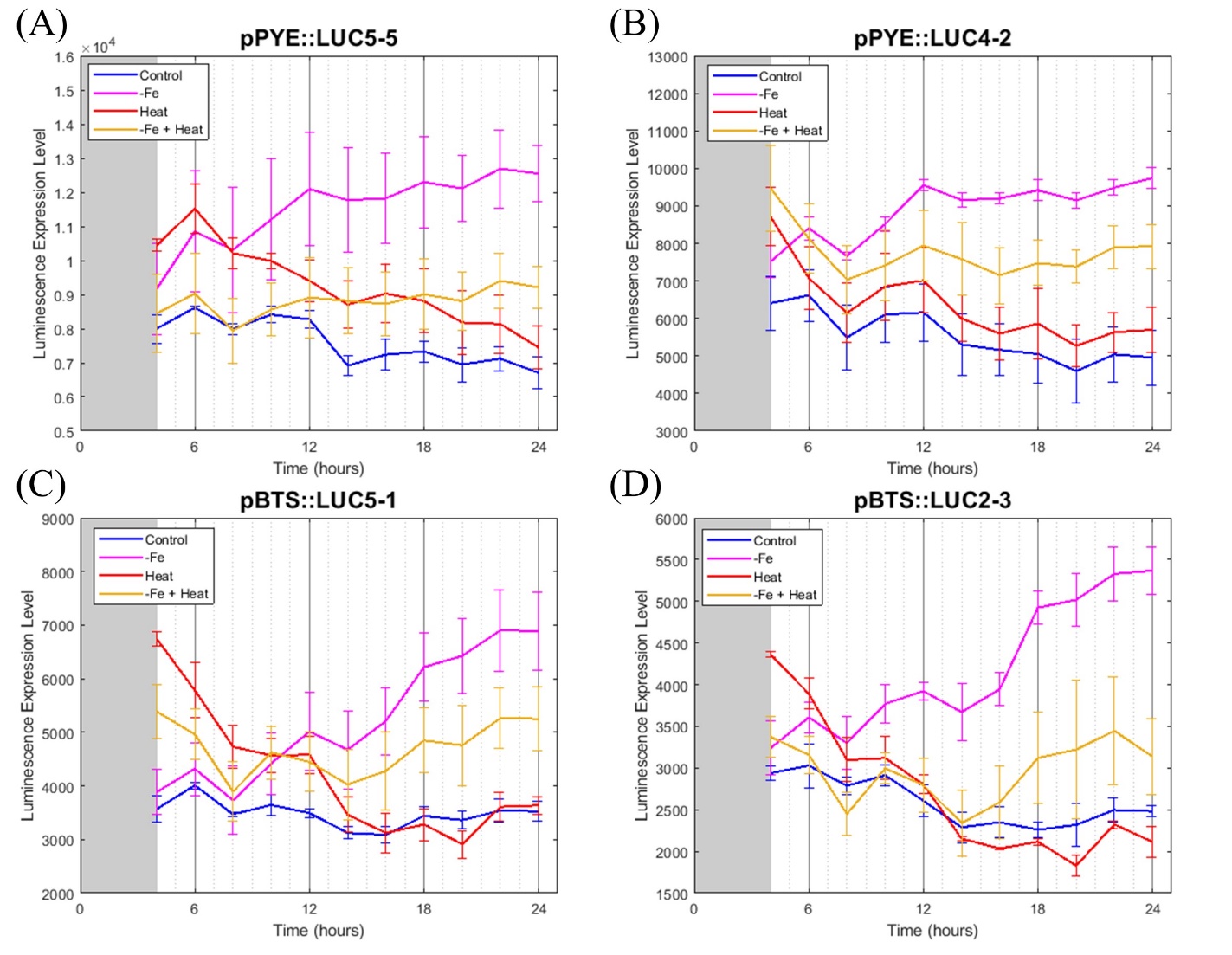


**Supplementary Figure 2.** Time course plots of PYE and BTS expression levels in *A. thaliana* measured by bioluminescence. Error bars show the standard error of n = 3 biological replicates. (A-B) Two different lines of pPYE::LUC *A. thaliana* seedlings showed to have similar temporal expression patterns of PYE measured by bioluminescence. The pPYE::LUC4-2 line is the same line that is shown in Figure 1 of the manuscript. (C-D) Two different lines of pBTS::LUC *A. thaliana* seedlings showed to have similar temporal expression patterns of BTS measured by bioluminescence. The pBTS::LUC2-3 line is the same line that is shown in Figure 1 of the manuscript.
